# Tibial condylar valgus osteotomy (TCVO) for osteoarthritis of the knee: 5-year clinical and radiological results

**DOI:** 10.1007/s00402-016-2609-3

**Published:** 2017-01-28

**Authors:** Ko Chiba, Akihiko Yonekura, Takashi Miyamoto, Makoto Osaki, Goji Chiba

**Affiliations:** 1 Department of Orthopaedic Surgery, Nagasaki University Graduate School of Biomedical Sciences, 1-7-1 Sakamoto, Nagasaki, 852-8501 Japan; 2 Department of Orthopaedic Surgery, Nishi-Isahaya Hospital, 3015 Kaizu, Isahaya, Nagasaki, 854-0063 Japan

**Keywords:** Osteoarthritis of the knee, High tibial osteotomy, Tibial condylar valgus osteotomy

## Abstract

**Purpose:**

Tibial condylar valgus osteotomy (TCVO) is a type of opening-wedge high tibial osteotomy for advanced medial knee osteoarthritis (OA) with subluxated lateral joint. We report the concept, the current surgical technique with a locking plate, and the short-term clinical and radiological results of this procedure.

**Methods:**

11 knees with medial OA and a widened lateral joint were treated by TCVO (KL stage III: 6, IV: 5). In this procedure, by the L-shaped osteotomy from the medial side of the proximal tibia to the intercondylar eminence and the valgus correction, lateralization of the mechanical axis and reduction of the subluxated lateral joint are obtained with early postoperative weight-bearing. Before, 6 months, 1, and 5 years after the operation, a visual analog scale (VAS), the Western Ontario and McMaster Universities Arthritis Index (WOMAC), alignment of the lower extremity, and congruency and stability of the femorotibial joint were investigated.

**Results:**

The VAS improved from an average of 73 mm to 13 mm, and the total WOMAC score from 52 to 14 before to 5 years after the operation, respectively. The mechanical axis changed from 1 to 60%, and the FTA changed from 186° to 171°. The joint line convergence angle (JLCA) changed from 6° to 1°, and the angle difference of JLCA between varus and valgus stress improved from 8° to 4° after the procedure.

**Conclusion:**

Improvements in pain and activities of daily living were observed by TCVO along with valgus correction of the lower extremity and stabilization of the femorotibial joint.

## Introduction

**Fig. 1:**
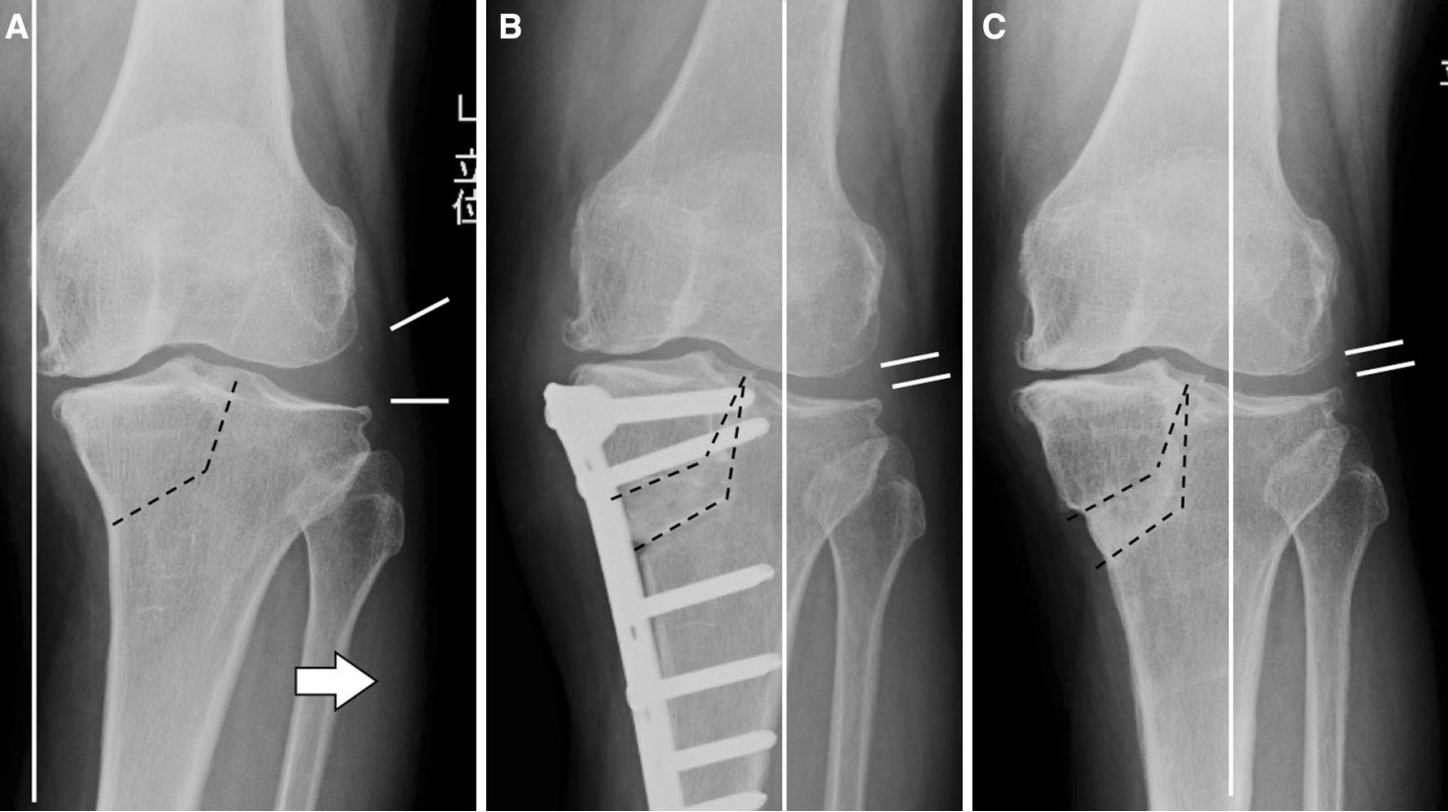
With an L-shaped osteotomy from the medial side of the proximal tibia to the intercondylar eminence and making the valgus correction (**a**), lateralization of the load line and definite reduction of the subluxated lateral joint are obtained (**b**, **c**)

High tibial osteotomy (HTO) is an effective treatment for unicompartmental varus knee osteoarthritis (OA), especially for young and elder patients as well as physically active individuals, despite the widespread use of joint replacements [1–5]. Tibial condylar valgus osteotomy (TCVO) is a type of opening-wedge HTO that was developed in 1990 in Japan [6]. By the L-shaped osteotomy from the medial side of the proximal tibia to the intercondylar eminence and by correcting the knee alignment from varus to valgus, TCVO alters the mechanical axis to lateral and reduces the subluxated lateral joint (Fig. 1).

**Fig. 2:**
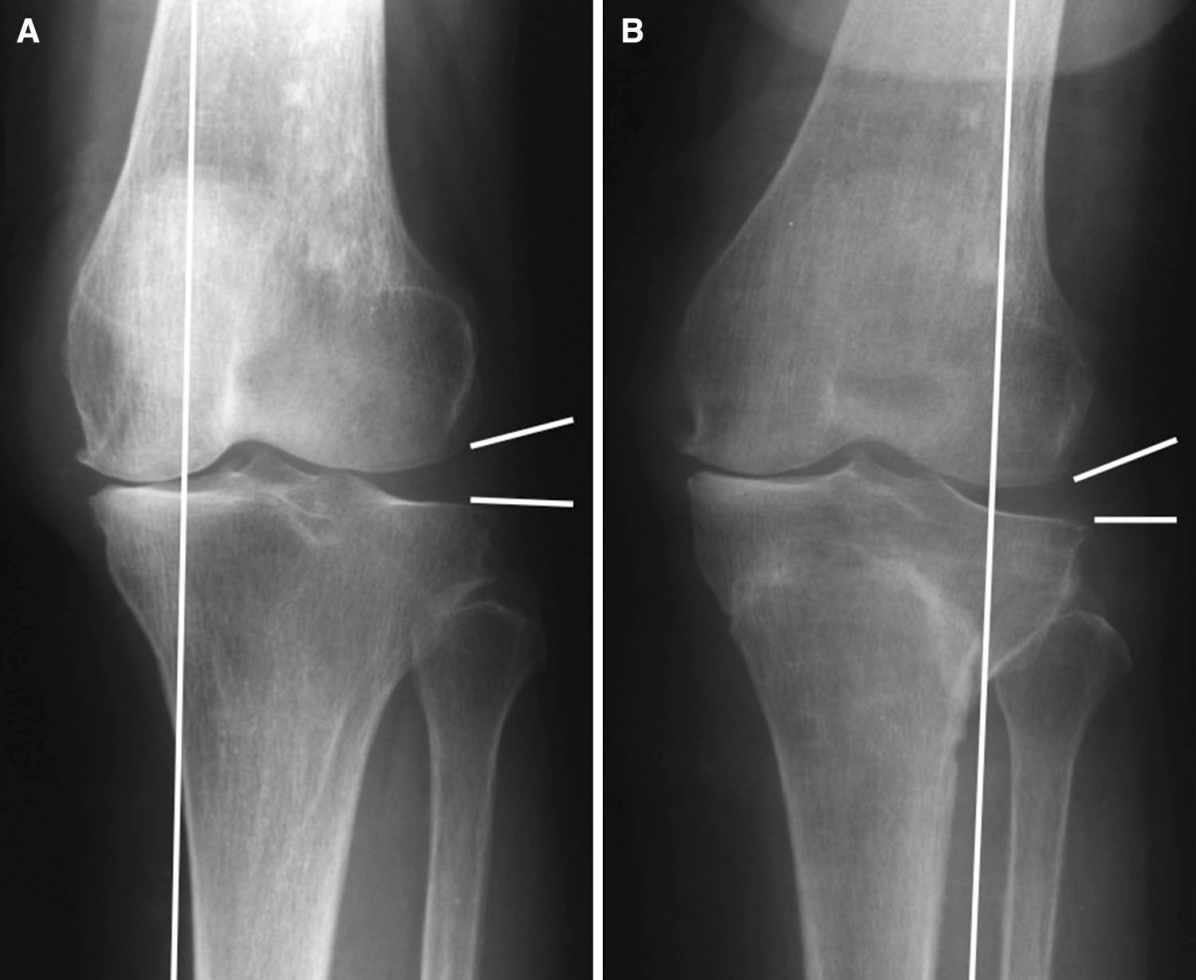
Advanced cases of varus knee OA have a dilated and subluxated lateral joint (**a**). Even when the mechanical axis has been moved to the lateral side by HTO, the lateral joint occasionally does not make contact (**b**), which means that the purpose of HTO, the reallocation of stress distribution, is not achieved

The background of the development of TCVO is that closing wedge or dome HTO was mainly performed during the 1980s and 90s, but there were some cases in which a lateral joint was not reduced despite the mechanical axis having been lateralized (Fig. 2). In an advanced case of varus knee OA, the stress is concentrated on the medial joint, while the lateral joint dilates until it becomes subluxed. Especially in such advanced cases, even when the mechanical axis has been moved to the lateral side, some cases have occasionally been found where the lateral joint does not make contact, which means that the purpose of HTO, which is the reallocation of stress distribution, is not achieved [7]. Therefore, the technique reported in this paper, which can provide not only a transfer of the mechanical axis but a reliable reduction of the lateral joint by applying an L-shaped osteotomy line to the intercondylar eminence, was proposed.

Due to improvements in implants in recent years, opening-wedge osteotomy using locking plates has become the main method for HTO [8, 9]. Simultaneously, TCVO is also now making use of locking plates, with shorter postoperative rehabilitation as a result. We hypothesized that TCVO with locking plate would provide satisfied clinical results for patients with advanced medial OA. In this report, the current operative procedures of TCVO are described, and its clinical and radiological results are reported.

## Methods

### Subjects

The subjects were 10 cases involving 11 knees that underwent TCVO in our institute from July 2008 to December 2009 (average age 57 ± 6 years, age range 49–65 years; 3 males 7 female, Kellgren–Lawrence stage III: 6, IV: 5) [10]. Height is 158.2 ± 9.5 cm (148–177.5 cm), weight is 72.9 ± 10.6 kg (61.6–86.4 kg), and BMI is 29.0 ± 2.7 kg/m^2^ (25.5–33.5 kg/m^2^).

The indication for TCVO was middle to end-stage medial unicompartmental OA with lateral joint dilation, with range of motion (ROM) >90° and flexion contracture <10°. Patients with lateral OA, excessive varus deformity of the tibia, lateral bowing of the femur, inflammatory arthritis, and smokers were excluded from the surgery.

We performed standard HTO in 5 patients (5 knees: KL II), and TCVO in 13 patients (14 knees: KL-III, IV) from July 2008 to December 2009. 3 patients (3 knees) could not be followed after TCVO because they live far from our hospital. Finally, 10 patients (11 knees) were investigated in this study.

This study was approved by the research ethics committee of our hospital and informed consent was obtained from all participants.

### Surgical procedure

A skin incision is placed at the anteromedial part of the proximal tibia, and then exposing the periosteum of the anteromedial osteotomy part. Posterior soft tissue is not stripped to minimize avascular bone necrosis.

The medial part of the L-shaped osteotomy is conducted with a chisel and bone saw under direct vision. The apex of the L-shaped osteotomy line is on the medial border the patellar tendon insertion on the tibial tuberosity. The osteotomy to the intercondylar eminence is implemented with a chisel under fluoroscopic guidance. In the AP view, a chisel is inserted toward the lateral beak of the intercondylar eminence cutting the anterior and superior cortical bones. Then the posterior cortical bone is cut looking the lateral view.

Valgus correction is performed aiming to achieve 65% of the mechanical axis [11]. The spreader is put at the posterior cortical bone to avoid the posterior tilt of tibia slope. Opening width of the osteotomy can be estimated preoperatively. The postoperative mechanical axis is lined on 65% and the center of rotation is put on the intercondylar eminence, then correction angle and opening width can be measured.

After sliding in a locking plate (Tomofix Japanese: Synthes, Bettlach, Switzerland) subcutaneously using minimally invasive plate osteosynthesis (MIPO) technique, the osteotomy is stabilized with locking screws. Screws do not have to be inserted deeply, but they should be inserted beyond the center of the proximal tibia. Artificial bone graft of β-TCP (OSferion: Olympus Terumo Biomaterials, Tokyo, Japan) is performed for any open space [12].

The operation time is about 90 min, and there is no need for blood transfusion. As for postoperative rehabilitation, full weight-bearing is allowed from the day after surgery depending on pain, and ROM exercise is also started at the same time.

### Clinical and radiological evaluations

As clinical evaluation, a visual analog scale (VAS), Western Ontario and McMaster Universities (WOMAC) score, and ROM before the procedure, a half-year after, 1, and 5 years after the procedure were evaluated. VAS is a pain score, and 100 mm indicates the severest pain. WOMAC is a score for pain, stiffness, and difficulty in activities of daily life. There are a total of 24 items, and each item is evaluated using 0–4 points, with 96 points being the worst score [13].

**Fig. 3:**
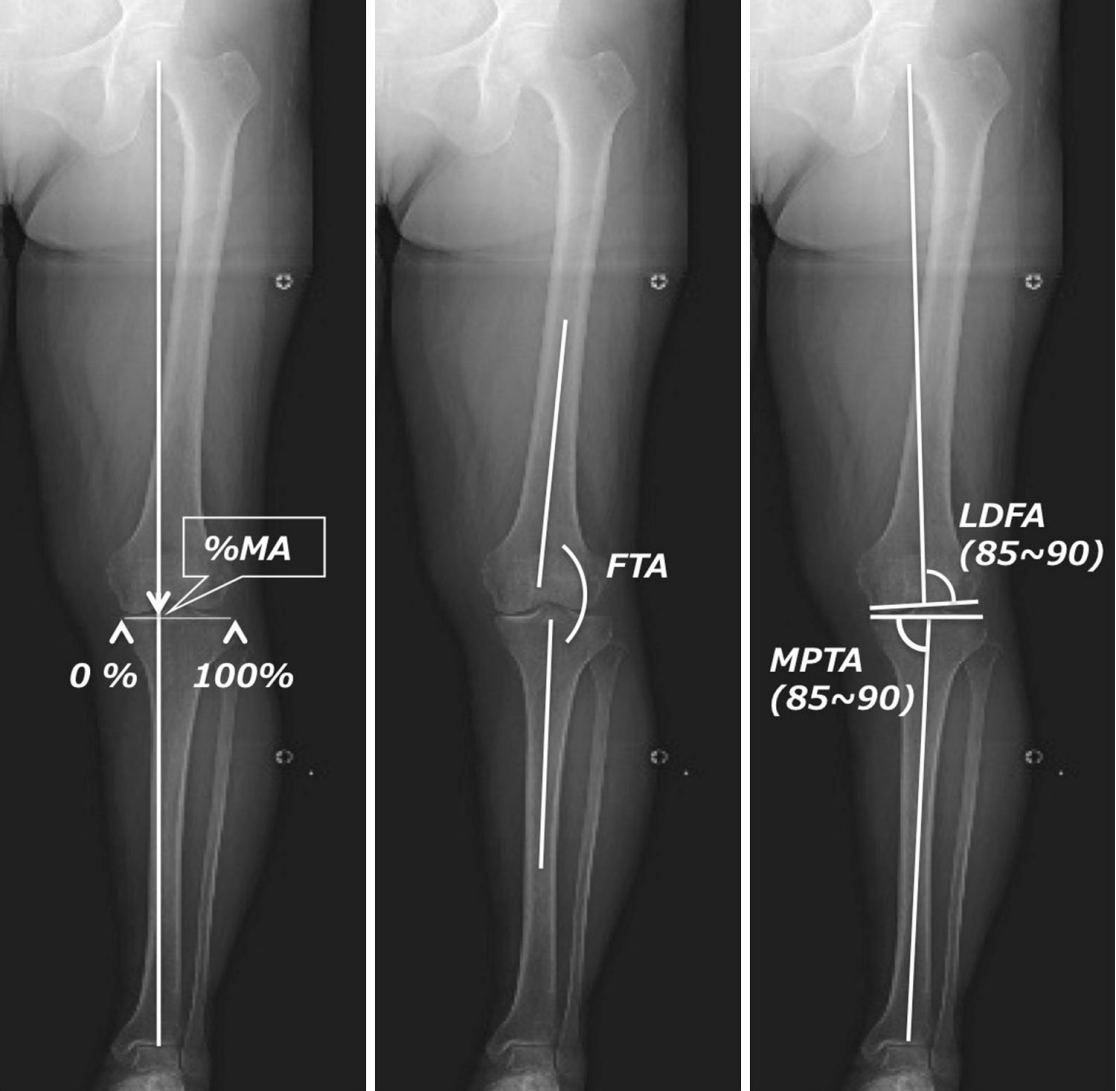
Mechanical axis (percentage of MA: %MA), femorotibial angle (FTA), lateral distal femoral angle (LDFA), and medial proximal tibial angle (MPTA) were measured to evaluate leg alignment

**Fig. 4:**
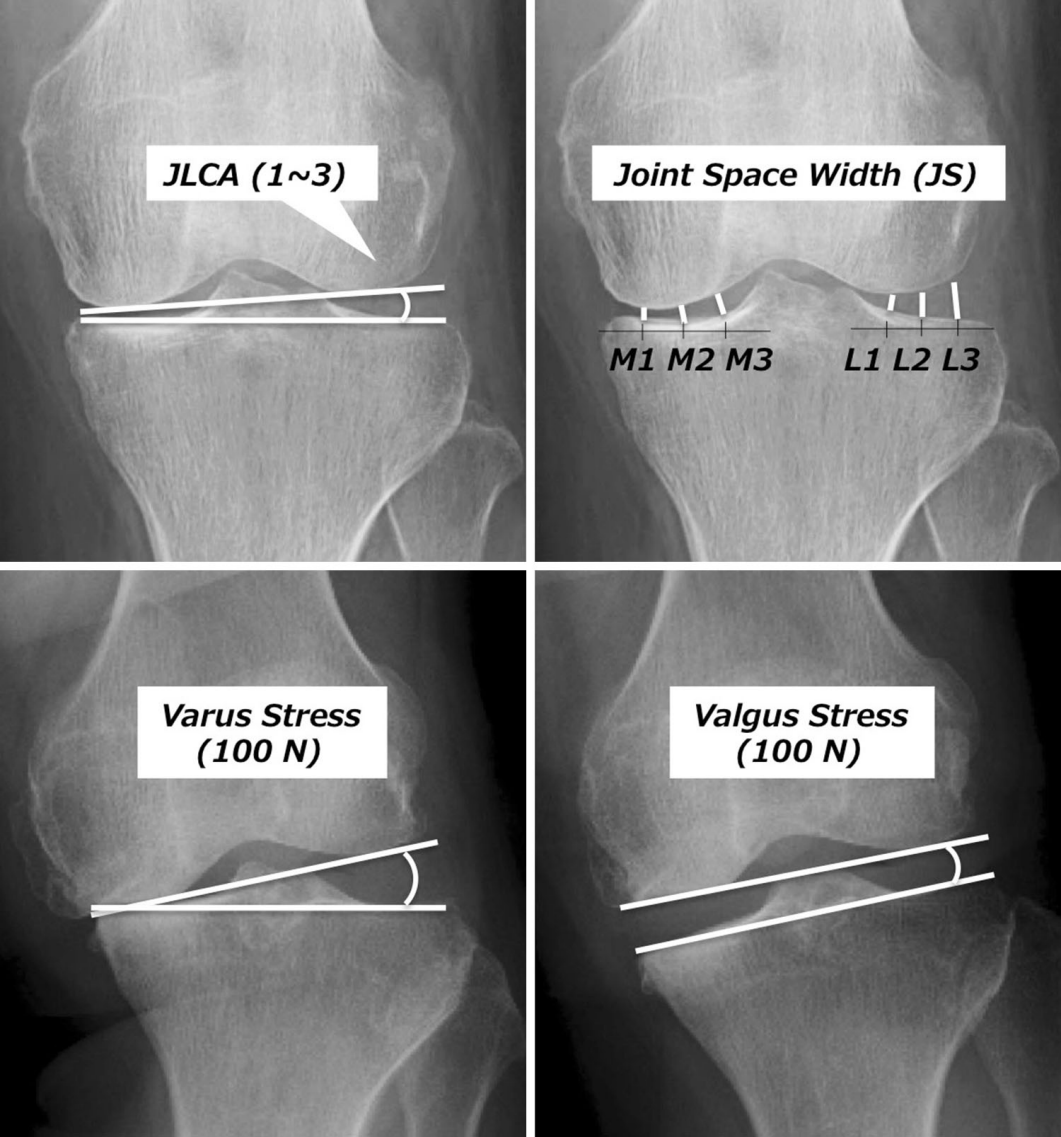
Joint line convergence angle (JLCA) and joint space width (JS) were measured to evaluate joint congruity. JLCAs with 100-N varus and valgus stress and their difference (ΔJLCA) were measured to evaluate joint stability

For radiological evaluations, an anteroposterior plain radiograph of the full-length legs in a standing position was performed. The mechanical axis (percentage of MA: %MA), femorotibial angle (FTA), lateral distal femoral angle (LDFA), and medial proximal tibial angle (MPTA) were measured to evaluate leg alignment (Fig. 3) [14, 15]. The joint line convergence angle (JLCA) and joint space width (JS) were measured to evaluate joint congruity. A positive JLCA value means a varus knee. JLCAs with 100-N varus and valgus stress and their difference (ΔJLCA) were measured to evaluate joint stability (Fig. 4).

As postoperative complications, the presence of fractures (medial or lateral compartment, avulsion of the intercondylar eminence), vascular or nerve damage, clinical deep vein thrombosis (DVT) and pulmonary embolism (PE), infections (superficial, deep), hardware issues, avascular bone necrosis, delayed or non-union, and loss or gain of correction were assessed.

### Statistical analysis

Statistical analysis was performed using SPSS Statistics ver. 21 (IBM Corp., Armonk, NY, USA). The results before, 1, and 5 years after the procedure were compared using the Wilcoxon test, and a *p* value of <0.05 was defined as significant.

## Results

**Fig. 5:**
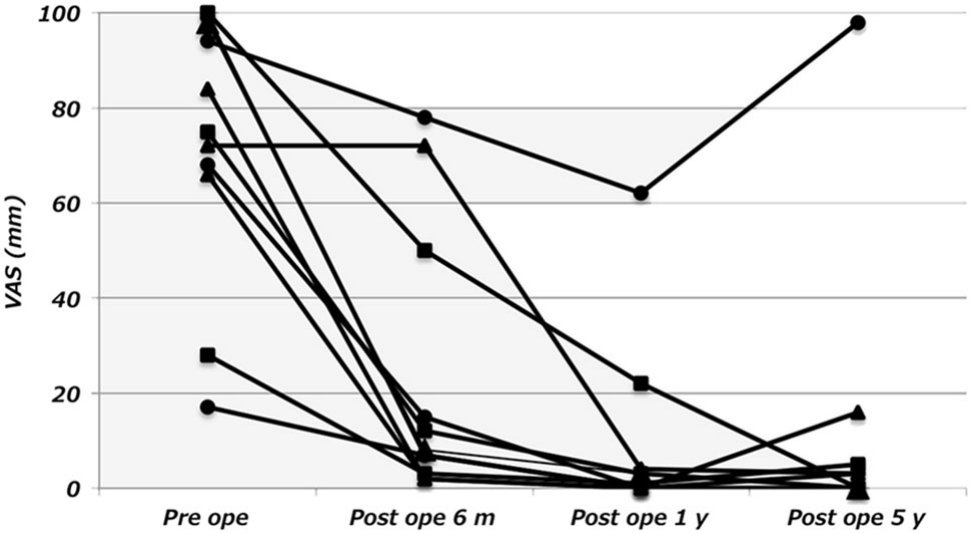
Individual changes of VAS before, 6 months after, 1 year after, and 5 years after TCVO. In most cases, VAS has improved 6 months after the procedure

VAS is showed in Table 1. The average VAS improved from 73 mm before the procedure to 9 mm 1 year after the procedure, and maintained to 13 mm 5 years after the procedure; in most cases, the VAS had already improved at 6 months after the procedure (Fig. 5). The pain score of WOMAC improved from 13 to 3, the stiffness score from 4 to 2, the daily activities score from 35 to 10, and the total score from 52 before the procedure to 14.5 years after the procedure. ROM before the procedure was −5° to 122°, while ROM 5 years after the procedure was −4° to 116°.

**Table 1:** VAS and WO MAC Index before and after TCVO

	Pre-op	Post-op 6 months	Post-op 1 year	Post-op 5 years	*p*_a_	*p*_b_
VAS (0–100 mm)	73 ± 28 (17–100)	23 ± 29 (2–78)	9 ± 19 (0–62)	13 ± 30 (0–98)	<0.01	<0.01
WOMAC (0–96)	52 ± 22 (16–79)	20 ± 19 (0–64)	11 ± 21 (0–73)	14 ± 26 (0–89)	<0.01	<0.01
Pain (0–20)	13 ± 4 (5–16)	5 ± 5 (0–14)	3 ± 5 (0–16)	3 ± 6 (0–19)	<0.01	<0.01
Stiffness (0–8)	4 ± 2 (2–7)	2 ± 1 (0–4)	1 ± 2 (0–5)	2 ± 2 (0–7)	<0.01	<0.05
Daily activities (0–68)	35 ± 17 (9–57)	13 ± 14 (0–48)	8 ± 15 (0–52)	10 ± 18 (0–63)	<0.01	<0.01

*VAS* visual analog scale, *WOMAC* Western Ontario and McMaster Universities score

Wilcoxon test: ^a^ pre-op and post-op 1 year, ^b^ pre-op and post-op 5 years

As seen in Table 2, average %MA improved from 1% before the procedure to 60% after the procedure, FTA improved from 186° to 171°, meaning that alignments of the lower extremities were corrected to valgus. There was no change in LDFA, whereas MPTA changed from 83° before the procedure to 91° after the procedure, meaning that the tibia was corrected to valgus.

**Table 2:** Radiological parameters before and after TCVO

	Pre-op	Post-op 1 year	*p*
%MA (%)	1 ± 19 (−32 to 27)	60 ± 15 (40 to 91)	<0.01
FTA (°)	186 ± 4 (179 to 194)	171 ± 3 (165 to 176)	<0.01
LDFA (°) (85–90)	88 ± 2 (86 to 91)	88 ± 1 (87 to 91)	ns
MPTA (°) (85–90)	83 ± 2 (81 to 87)	91 ± 4 (84 to 96)	<0.01
JLCA (°) (1–3)	6 ± 3 (3 to 10)	1 ± 2 (−3 to 4)	<0.01
JS: M1/M2/M3 (mm)	1.5 / 2.1 / 4.1	3.3 / 3.5 / 4.8	<0.05
JS: L1/L2 /L3 (mm)	3.1 / 6.4 / 8.5	4.3 / 5.3 / 5.3	<0.05
JLCA 100 N varus stress (°)	8 ± 2 (4 to 11)	4 ± 1 (1 to 6)	<0.01
JLCA 100 N valgus stress (°)	0 ± 1 (−2 to 2)	0 ± 2 (−3 to 2)	ns
ΔJLCA (°)	8 ± 2 (3 to 12)	4 ± 2 (2 to 7)	<0.01

Wilcoxon test between pre-op and post-op 1 year

%*MA* mechanical axis, *FTA* femorotibial angle, *LDFA* lateral distal femoral angle, *MPTA* medial proximal tibial angle, *JLCA* joint line convergence angle (85–90 and 1–3 means normal range), *JS* joint space width (only averages are shown)

**Fig. 6:**
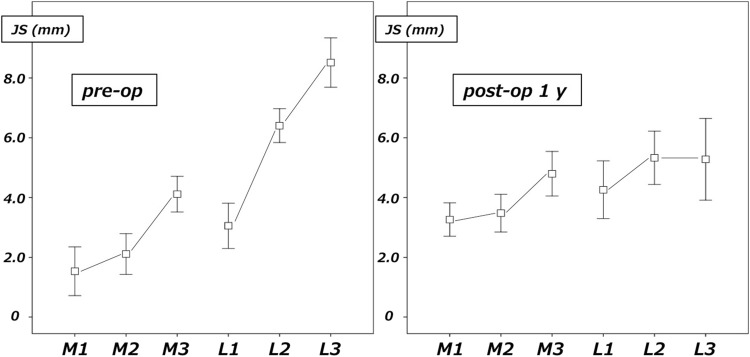
Joint space width before and 1 year after TCVO. The joint space is narrowed in the medial joint and widened in the lateral joints before the procedure. Not only the joint space narrowing of the medial joint but joint congruence of the lateral joints has improved after the procedure

JLCA changed from 6° before to 1° after the procedure, meaning that intra-articular valgus correction was also obtained. The JS of the medial joint before the procedure was narrowed to 1.5, 2.1, and 4.1 mm sequentially from the medial side, while those of the lateral joint were widened to 3.1, 6.4, and 8.5 mm. After the procedure, joint space narrowing of the medial joint improved to 3.3, 3.5, and 4.8 mm. In addition, joint congruence of the lateral joint improved to 4.3, 5.3, and 5.3 after the procedure (Fig. 6).

Before the procedure, JLCA with varus stress was 8° and that with valgus stress was 0°; thus, the angle difference was 8°. After the procedure, JLCA with varus stress was 4° and that with valgus stress was 0°, resulting in an angle difference of 4°, meaning that the joint instability between varus and valgus stress improved.

Posterior tilt angle of tibia slope is 82.8° ± 3.3° (79°–89°) preoperatively and 77.1° ± 2.5° (74°–81°) postoperatively. Average 5.7° of posterior tilt was obtained after surgery.

Generally, most patients can walk using a walker with full weight-bearing 1–7 days after surgery, and walk with one cane 1–3 weeks after surgery. 90° of ROM are obtained by 1–2 weeks’ rehabilitation.

As for postoperative complications, there were no fractures into the medial or lateral compartment, avulsion fractures of the intercondylar eminence, vascular damage, clinical DVT and PE, deep infections, hardware issues, avascular bone necrosis, or delayed or non-union of the osteotomy site. One case had slight paraesthesia around the proximal site of the skin incision. One case had a superficial infection around the distal site of the skin incision 3 months after the operation, although it improved with antibiotic treatment. One case had 3° of loss of correction, while one case had 4° of gain of correction. One case had cerebral infarction due to cardiogenic embolism 2 years after the operation. 5-year follow-up was not performed in this case because of aphasia and hemiplegia.

## Discussion

This is the first report describing the concept, the indication, the current operation technique, and the short-term results of TCVO with a locking plate. This procedure has been performed in Japan since 1990, [16] and it has recently received attention because of the spread of HTO using locking plates.

This study confirmed that TCVO created valgus alignment of the lower extremity with an increase of %MA and a decrease of FTA (Table 2). In addition, the normalization of the joint surface, which is the main concept of TCVO, was accomplished with a widened medial joint, a readjusted lateral joint, and a normalized JLCA (Table 2; Fig. 6). As a result, improvement of the joint instability in the coronal plane was confirmed, with decreased change of the angle by a varus–valgus stress test (Table 2).

Varus deformity of the lower extremity in knee OA can occur at three parts: lateral bowing of the femur (increased LDFA), varus deformity of the proximal tibia (decreased MPTA), and varus in the knee joint (increased JLCA). Most patients with medial knee OA have varus deformity at the proximal tibia. As the OA stage advances, varus also occurs in the knee joint [17]. Because standard HTO only manipulates the proximal tibia to the valgus position, it changes MPTA, yet it does not always change JLCA. On the other hand, TCVO can alter JLCA in addition to MPTA, making it suitable for cases with a large JLCA, a widened lateral joint.

The VAS was largely improved within 1 year after the operation, and the mean value was 9 after 1 year and 13 after 5 years (Table 1). However, one case had 62 of VAS after 1 year and 98 after 5 years, which increased the overall average (Fig. 5). The VAS except this case had an average score of 3 after 1 year and 3 after 5 years also. Although TCVO provided resolution of pain for most cases, some cases required more time for improvement or could not obtain satisfied result, and we thought that further ingenuity was needed to obtain stable results for these cases.

This procedure has recently been changing with the use of a locking plate with minimal invasion, artificial bone graft of β-TCP instead of autograft from the ilium, and allowing full weight-bearing the next day instead of partial weight-bearing 5 weeks after the procedure, thus drastically reducing the patients’ burden. With the development of the locking plate adapted for opening-wedge HTO (Tomofix Japanese: Synthes, Bettlach, Switzerland), the same plate is used for TCVO. Since the proximal tibia is fixed with four locking screws for angular stability, and the osteotomy is not extended to the lateral tibial condyle, early weight-bearing according to the pain level is allowed. Most patients can walk with a walker within a week after surgery. In addition, autogenous bone graft from the iliac crest is no longer necessary, and this eliminates the postoperative pain at the donor sites.

The advantages of TCVO are: (1) the lateral joint can be reduced during the operation; (2) joint instability can be improved; (3) less risk of hinge fracture; (4) no need for long screw insertion; and (5) early weight-bearing can be started.

TCVO enables us to correct not only leg alignment, but also the articular surface and joint instability. It can ensure reduction of a subluxated lateral joint, which permits us to confirm the load redistribution to the lateral joint during the operation. Adjustment of the excess space in the lateral joint can also improve the varus–valgus instability of the knee joint.

In the standard opening-wedge HTO, it is necessary to preserve the lateral cortical bone of the tibia. Bone fracture to the lateral cortical bone or the lateral compartment due to an inappropriate osteotomy can result in non-union or lateral OA [18, 19]. Furthermore, in the standard HTO, it is necessary to perform osteotomy in two planes to preserve the tibial tuberosity, and there is a risk of fracture of the tibial tuberosity. These major technical complications of HTO are not seen in TCVO.

When the screws are inserted, they need to be placed deep enough to reach the lateral part of the tibia to support the load in the standard HTO, but there is a possibility that they could penetrate the posterior cortex, not being long enough to support the load and damaging the posterior tissues. In TCVO, they only need to be inserted down to a little over the center of the tibia. Therefore, even if the plate is placed slightly anterior to the tibia, it is possible to insert screws with enough length.

In addition, because the osteotomy line of TCVO does not reach the lateral tibial condyle, it is possible to start early weight-bearing. With the valgus correction and lateralization of the mechanical axis, most of the load passes through the lateral tibial condyle where the osteotomy is not performed.

The disadvantage of TCVO is the limited angle of valgus correction. TCVO can correct the tibia to valgus only to the point that the lateral joint is reduced. Therefore, this procedure basically should not be applied for the patients without lateral joint subluxation. The clear indication is determined by preoperative planning, comparing lateral joint correctable angle with tibia correction angle needed for 65% of MA. For a severe case of tibia varus deformity, the lateral joint needs to be over-corrected so that the alignment of the lower extremity becomes valgus enough. Also, since soft tissue balance cannot be modified directly by this procedure, medial tightness and lateral loose may remain after the surgery.

The limitations of this study include the small number of cases and the short follow-up. We have started TCVO using locking plates and MIS technique since 2008, and these are data of our early series. We are planning to perform large-scale study over the long term. In addition, it is necessary to investigate the adverse prognostic factors and specify the indications for this procedure in more detail to stabilize the results. There is a possibility that the pathogenesis of knee OA is different between Asian female and Caucasian male patients. This procedure might not be applied to most of Caucasian male patients.

In conclusion, by performing TCVO, improvements in pain and activities of daily living were observed with valgus correction of the lower extremity along with reduction and stabilization of the femorotibial joint. With making early weight-bearing possible and a minimal risk of serious postoperative complications, the effectiveness of TCVO for varus knee OA with a subluxated lateral joint was confirmed.
